# Alginate-Based Platforms for Cancer-Targeted Drug Delivery

**DOI:** 10.1155/2020/1487259

**Published:** 2020-10-07

**Authors:** Lili He, Zhenghui Shang, Hongmei Liu, Zhi-xiang Yuan

**Affiliations:** ^1^College of Pharmacy, Southwest Minzu University, Chengdu 610041, China; ^2^Department of Orthopedics, The People's Hospital of China Three Gorges University, First People's Hospital of Yichang, Yichang 443000, China

## Abstract

As an acidic, ocean colloid polysaccharide, alginate is both a biopolymer and a polyelectrolyte that is considered to be biocompatible, nontoxic, nonimmunogenic, and biodegradable. A significant number of studies have confirmed the potential use of alginate-based platforms as effective vehicles for drug delivery for cancer-targeted treatment. In this review, the focus is on the formation of alginate-based cancer-targeted delivery systems. Specifically, some general chemical and physical properties of alginate and different types of alginate-based delivery systems are discussed, and various kinds of alginate-based carriers are introduced. Finally, recent innovative strategies to functionalize alginate-based vehicles for cancer targeting are described to highlight research towards the optimization of alginate.

## 1. Introduction

Cancer cells are believed to arise from the transformation of normal cells. Considering that anticancer drugs are typically toxic to both cancer and normal cells, undesirable side effects and minimal treatment efficiency, respectively, can result from an inability to discriminate between healthy and cancerous cells [1]. To specifically target and eradicate cancer cells, it is urgent to distinguish cancer cells from normal cells with high precision, such as the development of smart drug delivery platforms. To construct such smart platforms, suitable polymers should be chosen.

During the last decades, biodegradable polymers which can be classified into synthetic and natural polymers, depending on the source, have shown the most promising potential for building drug delivery systems (DDSs) for anticancer drugs [2, 3]. With a wide range of resources, natural polymers, especially polymers from marine organisms, are generally considered much safer than synthetic polymers because of their biodegradability and biocompatibility [4, 5]. Alginate, the most abundant marine biopolymer in the world, is a linear and anionic polysaccharide usually applied in developing cancer-targeted DDSs. The primary source of alginate is isolation from the cell walls and the intracellular spaces of several brown seaweeds around the world, such as *Laminaria hyperborea*, *Macrocystis pyrifera*, and *Ascophyllum nodosum* [6]. As mentioned above, the properties of alginate include remarkable biodegradability, low toxicity, chemical versatility, crosslinking capability, and pH sensitivity[7]. It can be easily modified to obtain derivatives with diverse structures, properties, functions, and applications, which makes alginate an ideal material for generating multitasking DDSs for cancer imaging and therapy [8]. A better understanding and improvement in the performance of alginate will have a profound impact on its competitiveness against synthetic polymers [9]. With the emergence of new applications of alginate and its derivatives for targeting strategies in recent years, it is necessary to update and build a more systematic review of alginate-based platforms for cancer-targeted drug delivery. Therefore, this review is based on a search of PubMed, Google Scholar, and the NIH database for English-language articles containing the following terms: “alginate”, “drug delivery”, “targeting/targeted therapy”, “cancer”, and “carriers” and we give a comprehensive and critical update of the applications of alginate as a potential platform for cancer-targeted delivery systems. We also discuss the continuous progress of scientific research in the DDS field with the aim of highlighting advantages and problems encountered so as to address research towards the optimization of alginate.

## 2. Chemical Structure and Properties of Alginate

**Figure 1:**
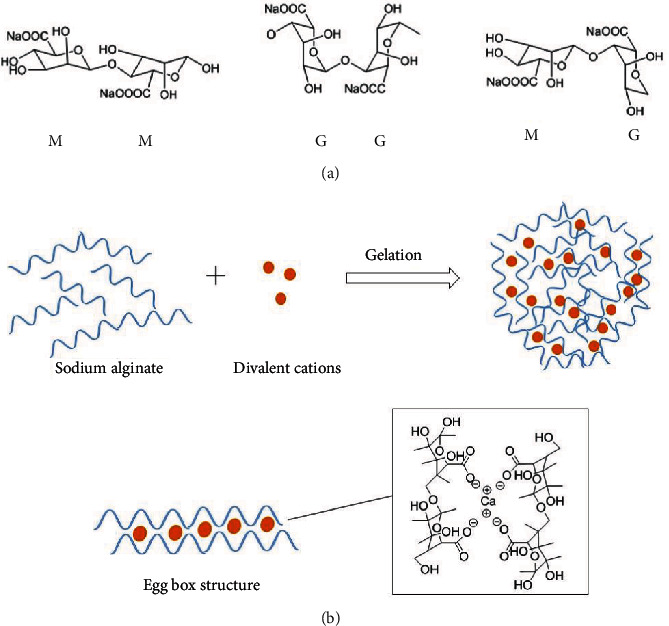
(a) Chemical structures of consecutive M segments, consecutive G segments, and alternating M and G segments. (b) Formation of alginate gel by divalent cations and egg box structure by ionic interaction of carboxylate ions of alginate G blocks and Ca^2+^.

Alginate is a polyelectrolyte, which is an anionic copolymer composed of 1,4-linked *β*-*D*-mannuronic acid (M) and *α*-*L*-glucuronic acid (G) units arranged in an irregular blockwise pattern [10]. The blocks are composed of consecutive G segments (GG), consecutive M segments (MM), and alternating M and G segments (GM) (Figure 1(a)) [11]. The M segments exhibit a flexible and linear conformation, while the G segments provide rigid and folded structural conformations that maintain the stiffness of the molecular chains [10]. Alginates isolated from different sources vary in both composition and block structure [12].

**Figure 2:**
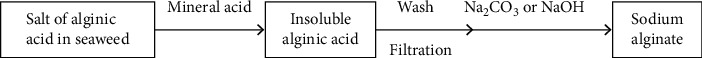
Schematic showing the procedure for alginate extraction from seaweed.

Alginate exists naturally as a mixed salt of cations that are found in seawater, mainly sodium, magnesium, and calcium ions [13]. To extract alginate from algae, mineral acid is usually used to remove the counterions and produce insoluble alginic acid, which is then solubilized by neutralization with an alkali such as sodium hydroxide or sodium carbonate to form sodium alginate [14] (Figure 2). Although another source to produce alginate is based on bacteria, such as *Azotobacter* and *Pseudomonas* species, this source is confined to small-scale research studies but is not viable for commercial applications [15].

A critical property of alginate is that, with certain crosslinking divalent cations, sodium alginate solution can undergo sol-to-gel transformation [15]. Gelation typically involves two mechanisms: external gelation and internal gelation. Other possible gelation methods are gelation by cooling, inverse, interfacial, and multistep interrupted gelation [16]. The gelation process involves the crosslinking of the alginate chain, caused by the exchange of sodium ions from the G blocks with divalent cations. The introduction of divalent cations makes the G blocks stack and then forms the characteristic egg box structure (Figure 1(b)). Each alginate chain can dimerize to form junctions with many other chains and then produce gel networks [17]. External crosslinking between cations and alginate polymer starts from the external surface of the droplet, which can produce thinner films with smoother surfaces, greater matrix strength, stiffness, and permeability than internally crosslinked films. Unlike external gelation, internal gelation starts its gelation from the core of the droplet[18]. Since it forms from the interior of the alginate droplet, it is also called as *in situ* gelation. External gelation seems to be a preferred method for producing crosslinked alginate for coating and drug encapsulation [18].

## 3. Methods for Constructing Alginate-Based Drug Delivery Platforms

Since alginate can easily be gelled with divalent cations under mild conditions, it is considered an ideal candidate for DDS applications. Various alginate platforms, especially those of micro- or nanosized, have been established for the delivery of chemical drugs produced by chemical synthesis or biosynthesis, genes, and proteins [19–21]. The drug loading and controlled-release performance of alginate-based DDSs can be easily adjusted via chemical modification or preparation techniques [22]. If necessary, their surface can be functionalized with groups or ligands to acquire specific functionality [23].

### 3.1. Nanogels

Nanogels are three-dimensionally crosslinked polymer networks that are composed of hydrogel particulate entities with a nanometer-sized space [24]. This platform combines the beneficial functions of larger hydrogel particles and those of nanosized particles, including high mechanical strength, the ability to prolong the circulation period of cargo loading in the bloodstream, and an enhanced permeability to tumor sites. In addition, it has large encapsulation cavities, the capability of swelling, and responsiveness so that it can easily be administered intravenously and deliver drugs to various target regions and cells [25]. Crosslinking is a necessary step to fabricate nanogels. The synthesis of nanogels is mostly achieved by two major strategies: the emulsion technique and the precursor technique [25, 26].

**Figure 3:**
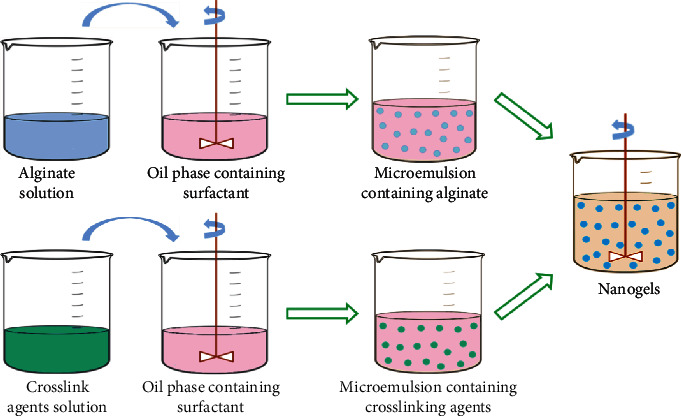
Schematic overview of the reverse microemulsion method for alginate nanogel preparation.

Sarika et al. prepared curcumin-loaded alginate aldehyde-gelatin nanogels with a hydrodynamic diameter of 431 ± 8 nm, a zeta potential of −36 ± 4 mV, and encapsulation efficiency of 72 ± 2% by applying the reverse microemulsion method [27]. In addition, it has been reported that oxidized sodium alginate with the aldehyde groups was synthesized to further prepare neutral protein-crosslinked nanogels (an average size of 150 nm) by simply adding CaCl_2_ solution into an oxidized sodium alginate solution under stirring [21]. The resulting oxidized sodium alginate nanogels would be disintegrated completely by removing Ca^2+^. In addition, other crosslinking methods contain hemoglobin and myoglobin, which are also used to crosslink oxidized sodium alginate into stable nanogels with the assistance of Ca^2+^ [21]. Furthermore, alginate nanogels could also be prepared by utilizing pluronic-based nanocarrier as a template and adding calcium ions to induce crosslinking inside the nanocarrier [28]. As a result, the formed nanogels maintain their stability for use in loading proteins and inducing sustained release. The affinity and ability of ionically crosslinked alginate gels are dependent on the type of cation used. Monovalent cations are another option for producing alginate gels. Podgorna et al. prepared gadolinium alginate nanogels with an average size of 110 nm using reverse microemulsions and a physical crosslinking method [22]. The process of the reverse microemulsion method is illustrated in Figure 3. Docusate sodium salt was used as anionic surfactant to generate stable microemulsion systems containing gadolinium and alginate, respectively. Then, two microemulsions were mixed, and gelation occurred inside the microdroplets, resulting in stable nanogels. In addition, active drugs can be used as crosslinking agents. Hong et al. successfully produced cisplatin-loaded alginate nanogels with a size of 100 nm [30]. The carboxylic acid groups of alginic acid were modified with iminodiacetic acid (IDA) to enhance the chelation of platinum ions. The formed nanogels had a narrow size distribution and stability. Furthermore, the cisplatin-loaded alginate nanogels showed highly pH-responsive drug release behavior and were capable of selective NIR fluorescence imaging and chemotherapy of macrophage cells. Another method for the preparation of pressure-sensitive 5-fluorouracil-loaded nanogels was described by Hosseinifar et al.[31]. Hydrogels were obtained via crosslinking of alginate with modified beta-cyclodextrin as a crosslinker, and then nanosized gels were produced by an emulsification method. 5-Fluorouracil was loaded into the nanogels in aqueous solution. The developed nanogels had an average particle size of 55.1 ± 5.1 nm with a noticeable drug encapsulation (82.1 ± 5.7%), which could induce a higher 5-Fu intracellular accumulation and a significant cell death extension by an apoptotic mechanism. It is believed that alginate-based nanogels can protect encapsulated drugs from degradation and provide a controlled release profile [31].

### 3.2. Microparticles and Microspheres

Microparticles or microspheres are commonly described as solid colloidal particles, ranging in size to 1 *μ*m [32, 33]. Crosslinking is essential in forming alginate microparticles or microspheres, despite the crosslinking agents and techniques, and additional materials might be needed. Yu et al. constructed alginate microparticles for protein encapsulation and controlled release [34]. A microfluidic approach was adopted for making uniform droplets with controlled size and size distribution using a specific device with four inlets and one outlet in which protein aqueous solutions, alginate solution containing CaCO_3_, and an oil phase were introduced. The pre-crosslinked alginate microparticles were formed in the microfluidic device and dropped into a gelation bath. The *in situ* and *ex situ* crosslinking strategies were combined to control the morphology of the alginate microparticles. Poly(ethyleneimine) and chitosan coating OVA-delta-insulin-encapsulated alginate microparticles improved protein retention efficiency, which can reach up to 90% and 80% protein encapsulation efficiency, respectively. Brassesco et al. also prepared alginate microparticles with an average size of 648 nm and zeta potential of -84 mV using a spray-drying technique and chemically crosslinking with epichlorohydrin [33]. The lysozyme and chymotrypsinogen protein adsorption capacity of the microparticles was demonstrated to be 1880 and 3034 mg/g, respectively, indicating alginate microparticles could be used for effective loading of protein. In addition, blue dextran could be used as a hydrophilic macromolecular model drug entrapped by alginate microspheres using a water-in-oil emulsion method [35]. The actual drug loading content and drug encapsulation efficiency of the crosslinked alginate microspheres were in the ranges of 6.98-7.43 wt% and 77-82%, respectively, which slightly increased as the Ca^2+^ concentration increased. Thus, alginate microparticles or microspheres are suitable carriers for hydrophilic drugs to obtain good loading and encapsulation efficiency.

Small molecular agents can also be entrapped into alginate microspheres. Freitas et al. used CaCl_2_ and PEG to crosslink sericin and alginate to form mucoadhesive sericin/alginate particles loaded with ibuprofen with a size range of 1.15 ± 0.4 mm to 3.15 ± 0.6 mm for sustained drug delivery [36]. The solution containing sericin/alginate and ibuprofen was dropped into a crosslinking agent solution to produce particles. The drug incorporation efficiency was improved by the addition of sericin and PEG, in the range of 73.01 ± 1.70% to 94.15 ± 4.21%. Alginate was found to exert an influence on the drug release, and the particles with the maximum alginate mass fraction showed sustained release through a dissolution mechanism. Hydroxyapatite/sodium alginate/chitosan (HA/SA/CS) composite microspheres were constructed using an emulsion crosslinking technique [37]. The doxorubicin (DOX) loading and encapsulation efficiency of HA/SA/CS composite microspheres were 46.86 ± 0.1414% and 93.72 ± 0.2828%, respectively, indicating that the three-dimensional network structure and rough surface of HA/SA/CS composite microspheres contributed to the improvement of the drug loading rate. Furthermore, DOX-loaded HA/SA/CS composite microspheres displayed good capability for pH-sensitive drug release, blood and cell compatibility, and better cell adhesion and proliferation capacity than the HA nanoparticles and HA/SA composite microspheres.

### 3.3. Nanoparticles

Nanoparticles can be defined as nanosized systems with diameters generally ranging from 10 to 1000 nm [38]. Nanosized DDSs have been widely recognized as having the potential to change pharmacokinetic profiles, reduce side effects, and enhance therapeutic efficiency[39, 40]. A type of calcium-alginate nanoparticles loaded with attenuated *Androctonus australis hector* (Aah) venom and its toxic fraction were fabricated as a vaccine delivery system [20]. The nanoparticles, with a sizes in the range 85–300 nm, were synthesized by adding CaCl_2_ solution to sodium alginate under constant homogenization and then freeze-dried without a cryoprotectant. The spray freeze-drying technique was used to prepare chitosan and alginate nanocomposite carriers intended for targeted colonic delivery, which were loaded with prednisolone and inulin [19]. Lertsutthiwong et al. prepared chitosan-alginate nanocapsules containing turmeric oil using the emulsion method [41]. Turmeric oil was emulsified in an aqueous sodium alginate solution, and the emulsion was gelled with CaCl_2_ and chitosan, followed by solvent removal. It was found that the characteristics of the nanocapsules were mostly dependent on the molecular weight and amount of chitosan. The average sizes of the chitosan-alginate nanocapsules with low molecular weight and medium molecular weight chitosan were 522 ± 15 and 667 ± 17 nm, respectively. Alginate/chitosan nanoparticles were to some extent able to stabilize and protect entrapped insulin from degradation in the gastrointestinal tract, which appears to be a promising trait for improving the oral absorption and oral bioactivity as an oral delivery system for insulin, and potentially for other therapeutic proteins [42, 43]. Therefore, freeze-drying, spray freeze-drying, emulsion, and complexation are standard methods for the production of alginate nanoparticles [19, 20, 41–43].

### 3.4. Aerogels

**Figure 4:**
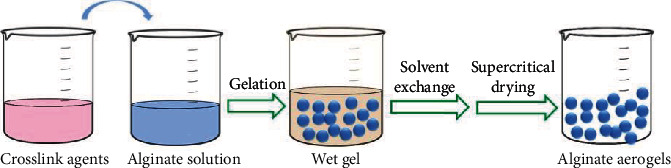
Processing scheme used for the preparation of alginate aerogels.

Aerogels are porous ultralight materials manufactured by using sol-gel chemistry. The liquid portion of the gel is replaced with gas without collapsing the solid network of the gel via a proper drying technology [44–48]. Aerogels exhibit favorable properties as the dispersed phase for drug delivery, including low density, high surface area, and high porosity with tunable surface chemistry [49, 50]. The process of preparing alginate aerogels usually involves three steps: solution, gelation, and drying [50–54], as illustrated in Figure 4. The first step is sol formation in which alginate and other materials are dispersed in an aqueous solution. Then, crosslinking agents are introduced to initiate the gelation point for building an interconnected three-dimensional network in the wet gel formation step. During the drying step, the liquid contained in wet gel is replaced by gas without the formation of a meniscus in the liquid-vapor interface, which causes a collapse of the entire structure [50]. Supercritical drying is usually adopted to eliminate the meniscus because the process is carried out at near-zero surface tension, and the liquid-vapor interface disappears, resulting in an aerogel instead of a xerogel [50, 55–59]. Therefore, blank alginate-based aerogels with a cylindrical shape were obtained using the supercritical drying method as follows: (1) alginate solutions were extruded in a coagulation bath of CaCl_2_ or CuSO_4_, where hydrogels were generated; (2) the corresponding aerogels were produced by supercritical drying at 200 bar and 45°C for 4 h [59].

**Figure 5:**
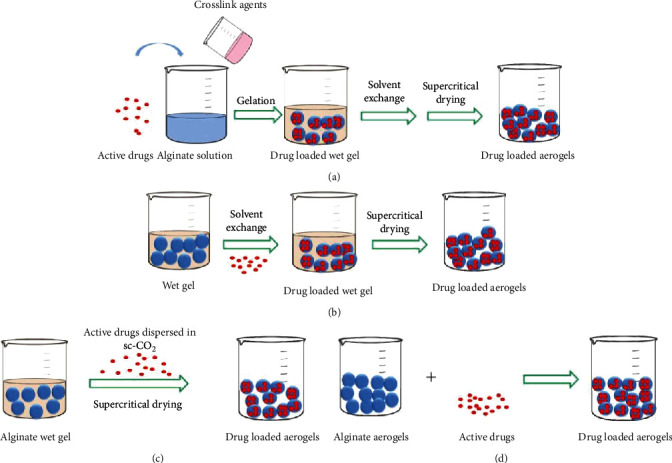
Schematic illustration of drug loading of alginate aerogels. (a) Loading before gelation. (b) Loading during the solvent exchange step. (c) Loading by supercritical deposition. (d) Soaking aerogels with drugs.

Several methods can be used for the entrapment of drugs into aerogels, including the addition of drugs before gelation or during the solvent exchange step, loading by supercritical deposition, and soaking aerogels with drug solutions which are primarily used [50, 60] (Figure 5). Considering the short stability of alginate hydrogels under dry air conditions, Veronovski et al. constructed a novel multimembrane onion-like alginate aerogel to enhance their stability [61]. They first prepared ionically crosslinked hydrogel spherical alginate cores, which were further immersed in alginate solution, dropped into the salt solution repeatedly, and converted into aerogels using supercritical drying. The model drug, nicotinic acid, was added to the alginate solution before crosslinking. Veres et al. prepared iron(III)-crosslinked alginate aerogel beads loaded with ibuprofen using the technique of adsorptive deposition from supercritical CO_2_ (sc-CO_2_) [62]. The aqueous alginate solution was dropped into an FeCl_3_ gelation bath and aged in FeCl_3_ solution for 24 h. After multiple-step solvent exchange process, the alginate gel beads were dried under supercritical CO_2_ at 45°C and 140 bars using a continuous flow process in a high-pressure autoclave to form aerogels. The loading of ibuprofen was carried out by impregnating the Fe(III)-alginate aerogel beads with ibuprofen, and a drug loading of 36-41 wt% was achieved. It was reported that an emulsification and internal setting technique were used to prepare coated and uncoated hybrid silica/alginate aerogel beads for controlled drug delivery [63]. A water phase containing tetramethyl orthosilicate (TMOS) and sodium alginate aqueous solution was dispersed into an oil phase to achieve a water-in-oil (W/O) emulsion to obtain a hybrid hydrogel dispersion. Water was then added to achieve phase inversion in the emulsion and to partition the hydrogel beads. The hydrogel beads were recovered and underwent solvent exchange. The drug was loaded before supercritical drying by suspending the alcogels in the ketoprofen solution, followed by supercritical drying. Goncalves et al. prepared alginate-based hybrid aerogel microparticles (<50 *μ*m) who employed similar water-in-oil emulsion gelation combined with the internal setting method [64]. It has been revealed that the addition of acetic acid microemulsion during the hydrogel formation step is a crucial step for minimizing particle agglomeration. At the same time, two methods for drug loading were studied: adsorption from sc-CO_2_ and adsorption by supercritical antisolvent precipitation. Loading of ketoprofen was performed by exposing the aerogel microparticles to the saturated solution of the drug in sc-CO_2_, whereas the loading of quercetin was performed during the final solvent exchange step because of its low solubility in sc-CO_2_. However, it seems that the production of aerogels with controlled pore size and dual pore size distribution still remains a challenge, which many efforts should be made to overcome.

### 3.5. Micelles

Micelles are formed by the self-assembly of amphiphilic molecules, which can generally generate nanosized organized core-shell structures in aqueous media at concentrations exceeding their critical micellar concentrations (CMC) [65–68]. Polymeric micelles are effective for drug delivery as they can selectively accumulate in solid tumors, and have improved loading capability, better therapeutic efficacy, and superior targeting ability following surface modification [69–72]. The cores of the micelles act as a reservoir for drugs, while the shell provides the required colloidal stability and avoids opsonization and protein adsorption *in vivo* [72]. Polymeric micelles can be roughly divided into three types in terms of their formation mechanisms: block copolymer micelles, graft copolymer micelles, polyelectrolyte micelles, or hybrid polyion complex micelles [73–77]. Since alginate is highly hydrophilic, hydrophobic modification is usually needed to form amphiphilic copolymers for micelle preparation. Yu et al. not only synthesized an alginate-g-poly(N-isopropyl acrylamide) (SA-g-PNIPAM) copolymer but also prepared thermosensitive hybrid polymer complex micelles in aqueous solutions by electrostatic interactions [77]. The core comprises a network of metal ion crosslinked sodium alginate chains, which is stabilized by the thermosensitive shell of hydrophilic PNIPAM chains [77]. Different metal ions caused the differences in the micelle diameters, which were in the ranges of 200-300 nm, 50-100 nm, and 30-60 nm for Ba^2+^, Zn^2+^, and Co^2+^ crosslinked micelles, respectively. Subsequently, 5-fluorouracil was loaded as a model drug by dissolving it in the polymer solution before crosslinking. The cumulative release of 5-fluorouracil from micelles was controlled by pH, ionic strength, or temperature of the surroundings. Furthermore, Sarika et al. first designed galactosylated alginate-curcumin conjugates to self-assemble into micelles with diameter of 235 ± 6 nm for enhanced delivery of curcumin to hepatocytes [78]. The self-assembly mechanism underlies the fact that the highly hydrophobic curcumin attached to the hydrophilic alginate chain introduces amphiphilic character and the tendency to form micelles in aqueous media (curcumin as the inner core and alginate as the outer shell). Using a similar alginate-curcumin conjugate, Lachowicz et al. prepared stable micelles (~200 nm) for anticancer applications [79]. The obtained data showed that the micellar structures formed by the alginate-curcumin conjugate were not mechanically strong and stiff enough and that the addition of calcium chloride to crosslink the alginate chain is indispensable. In addition, alginates could also be modified with hydrophobic materials to gain amphiphilic properties and, therefore, to form polymeric micelles by self-assembling [80, 81]. Hydrophobically modified alginate was synthesized by derivatization of sodium alginate with dodecyl glycidyl ether in an aqueous solution [80]. The prepared micelles had a spherical shape and good structural integrity with a size of approximately 1000-5000 nm and zeta potential of approximately -82.85 mV. Using Sudan IV as a hydrophobic model drug, it was demonstrated that the micelles possessed desirable drug loading properties. Among the micelles mentioned, the complex hybrid micelles based on alginate exhibited better stability and higher drug loading rate, which has gained increasing attention owing to their advantages.

## 4. Functionalization Strategies of Alginate-Based Platforms for Cancer Targeting

**Figure 6:**
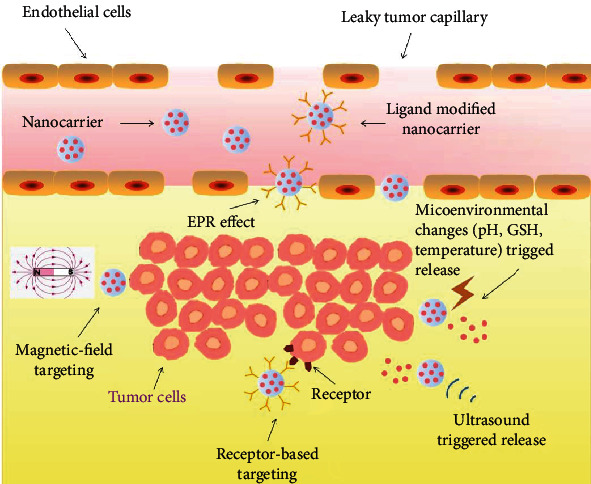
Various mechanisms for alginate-based nanocarriers to target cancer cells. Nanocarriers passively accumulated at the tumor site due to the EPR effect. Enhanced uptake of functionalized nanocarriers by cancer cells occurs via receptor-mediated endocytosis, microenvironmental stimuli-triggered release, and external physical stimuli-responsive targeting.

Targeted DDSs for cancer therapy are expected to enhance therapeutic efficacy with minimized side effects, since they can accumulate at the tumor site and discriminate small differences between healthy and cancerous cells [82–86]. The strategies for cancer-targeted drug delivery can be roughly divided into three types: passive targeting, active targeting, and stimuli-responsive release (Figure 6). The enhanced permeability and retention (EPR) effect, known as passive targeting, allows nanosized carriers to be distributed explicitly in the tumor at high concentrations and taken up by cells more efficiently [87]. Conjugation of ligand-receptor, antigen-antibody, and other forms of molecular recognition onto DDSs to obtain targeted delivery to specific cells, tissues, or organs is known as active targeting [88, 89]. Stimuli-responsive DDSs usually involve a phase transition in response to the microenvironmental changes of cancer cells such as temperature, pH, or a specific ion [83]. Alternatively, drug release could be triggered by externally noninvasive physical triggering signals, including ultrasound, heat, magnetic fields, and light [90]. Active targeting and stimuli-responsive release processes occur only after the passive accumulation of drug carriers in tumors, which is usually achieved by the nanosized carriers via the EPR effect. Thus, DDSs in nanosize are commonly constructed for cancer-targeted therapy. Herein, nanosized alginate-based cancer-targeted DDSs are summarized in Table 1.

### 4.1. Passive Targeting

In the passive targeting strategy, nanocarriers can extravasate through leaky tumor capillary fenestrations, resulting in their accumulation and retention[107]. Different types of nanoparticles can accumulate in other tissues or organs with distinct physiological properties in the body, showing high anticancer efficiency *in vitro* or *in vivo* by EPR or enhanced cancer cellular uptake effects when nanosized alginate-based carriers are fabricated. DOX-loaded poly(lactic-coglycolic acid) nanoparticles coated with chitosan/alginate were prepared using the layer-by-layer method [93]. *In vivo* studies demonstrated that the formed complex nanoparticles (diameters below 200 nm) had superior sarcoma tumor inhibition rates at 83.17% and faint toxicity, compared with uncoated DOX nanoparticles and free DOX. Employing a water-in-oil emulsification technique, Rosch et al. prepared DOX-loaded alginate/chitosan nanoparticles (~80 nm) [92]. It was shown that the nanoparticles were rapidly taken up by 4T1 murine breast cancer cells and produced high enough concentration to induce a therapeutic effect *in vitro*. Sorasitthiyanukarn et al. encapsulated curcumin diglutaric (CG) acid into chitosan/alginate nanoparticles [91], which gained better stability and slower release following a Weibull kinetic model compared with CG. The nanoparticles exhibited higher *in vitro* cellular uptake in human epithelial colorectal adenocarcinoma (Caco-2 cells) and higher anticancer activity against Caco-2, human hepatocellular carcinoma (HepG2), and human breast cancer (MDA-MB-231) cells. Furthermore, Mirrahimi et al. coloaded cisplatin and gold nanoparticles (AuNPs) into alginate hydrogel networks, forming the ACA nanocomplex [108]. Cisplatin is a commonly used anticancer agent, while AuNPs can function as radiosensitizers to enhance radiation-induced damage. Therefore codelivery is expected to amplify the efficacy of chemoradiation. *In vivo* data suggested that the ACA nanocomplex significantly improved the chemotherapy efficiency and yielded a 79% growth inhibition in CT26 colon adenocarcinoma tumors, compared to 9% for free cisplatin administration. The combination of the ACA nanocomplex with 6 MV X-rays showed a 51% enhancement in antitumor activity compared with standard chemoradiation. From the recent reports mentioned above, it was found that the design of passive targeting carriers based on alginate is complicated, and alginate combined with other functional materials could generate better passive targeting profiles compared to alginate alone.

### 4.2. Receptor-Based Targeting

#### 4.2.1. Folic Acid (FA) Receptor-Based Targeting

FA is a low-molecular-weight (441 Da), stable, inexpensive, and poorly immunogenic chemical with a high affinity for the folic acid receptor (FR) [109, 110]. Furthermore, FR is overexpressed on the surface of many types of human cancerous cells, but they are present in low or no detectable levels on most healthy cells. Typically, FA is conjugated to materials or nanoparticles by esterification, which occurs with high reactivity in the carboxylic acid portion of the secondary carbon in folic acid. Through layer-by-layer deposition, poly(lactide-coglycolide)-coated nanoparticles(~200 nm) and chitosan/alginate nanoparticles were prepared and covalently bonded to FA or FA-grafted PEG via carbodiimide chemistry [94]. *In vitro* studies suggested that the alginate-covered surface reduced particle attachment to HepG2 cells and cellular uptake. In contrast, the FA or PEG-FA modification on the surfaces of the carriers could increase both the uptake ratio and the number of nanoparticles per cell. To further investigate this possibility, Martino et al. prepared FA-chitosan-alginate nanocomplexes (70-120 nm) for codelivery of temozolomide and DOX [106]. *In vitro* studies suggested that in NIH/3T3 cells (which do not express FR), the presence of FA conjugation in the nanocomplex did not affect the cell viability, while in the HeLa cells (cells that overexpress FR), a clear difference in the inhibition by the unmodified and the FA-modified nanocomplexes was observed, which confirmed the importance of FA modification in ameliorating tumor cell uptake and selectivity. Pei et al. designed alginate-based cancer-associated, stimuli-driven, and turn-on theranostic prodrug nanogels (~250 nm) for tumor diagnosis and chemotherapy [100]. The authors crosslinked the folate-terminated poly(ethylene glycol) (PEG) and rhodamine B-terminated PEG-modified oxidized alginate (OAL-gPEG-FA/RhB) with cystamine, and covalently conjugated DOX via an acid-labile Schiff base bond. The obtained *in vitro* data indicated that the viability of HepG2 cells decreased to 46% with the nanogels at a concentration of 100 *μ*g/mL. By incubating HepG2 cells with different concentrations of the nanogels in the presence of FA, the viability of the HepG2 cells was higher than those incubated without FA, indicating that the free FA had a competitive interaction with the overexpressed FR in tumor cells and that the nanogels had a FR-mediated targeting function. Recently, novel alginate-conjugated folic acid nanoparticles (AF NPs) were prepared by conjugating alginate with folic acid, followed by encapsulation of 5-aminolevulinic acid through a water-in-oil (W/O) emulsion method. Enzymes or other external factors did not degrade the obtained FA NPs before reaching cancer cells, and fluorescent precursors were precisely and accurately delivered to cancer cells for cancer-specific fluorescence imaging [111]. Indeed, there are many biopolymer drug delivery systems conjugated with folic acid as a ligand to realize cancer specificity. Among them, studies based on alginate/chitosan and FA systems are appealing because of the lower cost and more straightforward conjugation of folic acid.

#### 4.2.2. Asialoglycoprotein Receptor- (ASGPR-) Based Targeting

ASGPR is primarily expressed on hepatocytes and facilitates internalization by clathrin-mediated endocytosis and exhibits a high affinity for carbohydrates specifically galactose, N-acetylgalactosamine, and glucose [112]. Since ASGPR on hepatoma cells can specifically bind with ligands containing *β*-*D*-galactose and N-acetylgalactosamine residues, galactosyl moieties could be utilized for functionalization in hepatocyte-targeted delivery systems [113, 114]. Galactosylated alginate-based carriers are expected to promote the uptake of therapeutic agents into hepatocellular carcinoma cells via ASGPR-mediated endocytosis.

To overcome these shortcomings and to improve the cancer therapeutic index of curcumin, Sarika et al. synthesized a galactosylated alginate-curcumin conjugate (LANH2-Alg Ald-Cur) for targeted delivery of curcumin to hepatocarcinoma cells [78]. Polymer-drug conjugates can easily self-assemble into micelles in an aqueous environment with a hydrophobic core arising from the organization of the hydrophobic curcumin moieties and a hydrophilic shell of galactosylated alginate or alginate. LANH2-Alg Ald-Cur micelles exhibited improved selective toxicity toward HepG2 cells *in vitro* compared with Alg-Cur, demonstrating the contribution of the galactose moiety. The authors also investigated the cellular uptake of the galactosylated and nongalactosylated conjugates. They showed that the presence of a galactose moiety enabled LANH2-Alg Ald-Cur micelles to be internalized into HepG2 cells. Insoluble drugs are insufficient to provide antitumor activity. The principal aim of grafting insoluble drugs to hydrophilic alginate is to magnify their solubility in aqueous media, enhance their activity, and promote the drug loading rate.

#### 4.2.3. Glycyrrhetinic Acid- (GA-) Based Targeting

GA and glycyrrhizin (GL), the main bioactive compounds extracted from licorice, are widely used in medicine for the treatment of many diseases [115–117]. There are specific binding sites for GL and GA on the cellular membranes of hepatocytes, and the number of binding sites for GA is much higher than that for GL [118]. Alginate-based carriers modified with GA are expected to have high accumulation in the liver and superior targeting efficiency to hepatocytes.

DOX has been demonstrated to be one of the most effective anticancer agents available at present. However, the wider clinical application of DOX is limited because of the toxic side effects such as myelosuppression and cardiotoxicity. Zhang et al. prepared the DOX-loaded glycyrrhetinic acid-modified alginate nanoparticles (DOX/GA-ALG NPs) for liver tumor targeting drug delivery and the average diameter of approximately 274.2 nm, where the degree of substitution of GA conjugation was 13.6 wt% [96]. Tissue distribution studies demonstrated that the concentration of DOX in the liver after the administration of DOX/GA-ALG NPs was about 5-fold higher than that of free DOX. At the same time, *in vivo* studies suggested that DOX/GA-ALG NPs produced a superior antitumor effect against mice bearing H22 orthotopic liver tumors without any apparent negative impact on normal liver tissue. The authors further fabricated DOX/GA-ALG NPs and investigated the biodistribution of the nanoparticles in mice as well as their antitumor efficiency and side effects *in vivo* [119]. The biodistribution data showed that the concentration of DOX in the liver reached 67.8 *μ*g/g after intravenous administration of DOX/GA-ALG NPs, which was 2.8-fold and 4.7-fold higher than that of non-GA-modified nanoparticles and free DOX, respectively. Histological examination revealed tumor necrosis in both experimental groups, as well as myocardial necrosis and apparent liver cell swelling in the free DOX group. There are two main reasons for these results: (1) there was a difference in the pH between tumor tissue and normal tissue. (2) DOX release from DOX/GA-ALG NPs was increased in the tumor microenvironment (pH 5.8), contributing to its enhanced antitumor activities. Another possible reason could be that the amount of GA receptor was different between liver tumor cells and normal liver cells.

#### 4.2.4. Epidermal Growth Factor Receptor- (EGFR-) Based Targeting

The EGFR tyrosine kinase family includes EGFR (HER1), HER2, HER3, and HER4 proteins, which generally trigger a complex signal transduction network controlling cell proliferation, differentiation, adhesion, and apoptosis [120]. Their high levels of expression in many epithelial tumors cause significant differences in the number of receptor molecules on the surface of malignant and healthy cells [121]. EGFR is aberrantly activated by various mechanisms and is associated with the development of a variety of tumors [121, 122]. Zhang et al. designed EGF-modified cisplatin-alginate conjugate (CS) liposomes for targeted delivery to EGFR-positive ovarian cancer cells [102]. They synthesized a cisplatin-alginate conjugate by conjugating cisplatin to the carboxylate end groups on sodium alginate. They fabricated EGF-modified CS liposomes (CS-EGF-Lip) (110 nm) using the thin film hydration method. Compared with free cisplatin or CS-PEG-Lip, specific cellular uptake and penetration in tumor spheroids *in vitro* were significantly enhanced with CS-EGF-Lip. CS-EGF-Lip significantly suppressed the proliferation and migration of tumors compared with free cisplatin. *In vivo* xenograft experiments revealed that the administration of CS-EGF-Lip enhanced the delivery of cisplatin into ovarian tumor tissues, leading to improvement of the antitumor efficacy while reducing nephrotoxicity and body weight loss in mice. Therefore, EGFR ligand-modified alginate-based platforms could specifically target EGFR-expressing tumors via receptor-mediated endocytosis, thereby increasing anticancer efficacy.

#### 4.2.5. Biotin Receptor-Based Targeting

Biotin (vitamin H) is a desirable tumor-targeted ligand due to the overexpression of its receptors in many cancer cells, such as ovarian cancer cells, colon cancer cells, lung cancer cells, kidney cells, and breast cancer cells. In contrast, the biotin receptor is rarely expressed in normal cells [123, 124]. Because biotin is an essential micronutrient, rapidly proliferating malignant cells require extra biotin receptors to meet their biotin uptake requirements [125]. A dual targeting vector was designed as a nano-in-micro structure based on entrapping biotin-modified micelles into alginate microparticles (AlgBioPf-M) [126]. These DDSs consist of two different targeting sections: alginate-based microscale carriers with an enteric targeting function, and biotin-attached docetaxel-loaded nanomicelles. Compared with free docetaxel, *in vivo* studies indicated that docetaxel-loaded Alg-BioPf-M had 27.4-fold higher bioavailability and achieved superior tumor inhibition of 84.6% against sarcoma 180 tumors. Thus, this specific overexpression of biotin receptors on tumor cells has been explored to develop biotin-conjugated alginate DDSs for cancer-targeted delivery of drugs.

### 4.3. Stimuli-Responsive Targeting

Since anticancer drugs are toxic and have serious side effects, ideal cancer-targeted DDSs are expected to provide secure encapsulation of the drugs before reaching the cancer site without leakage. Still they should be able to release the drug cargo after entering cancer tissues [127]. Although alginate-based DDSs can selectively accumulate in the target site via passive or active targeting, some strategies are needed to disassemble the DDSs after entering the cancer tissues. Stimuli-responsive DDSs can take advantage of the specific microenvironmental changes in tumors such as a harsh redox environment, acidic condition, and certain types of enzymes, and release their cargo in desired sites [128–130]. Alternatively, they can be designed to respond to externally applied physical stimuli such as temperature, ultrasound, electric fields, magnetic field, and X-rays [129].

#### 4.3.1. Thermoresponsive Targeting

Thermoresponsive targeting DDSs usually involve a particular type of stimuli-responsive polymer characterized by a temperature-dependent volume phase transition [131^]^. These polymers exhibit a transition at a temperature defined by a lower critical solubility temperature (LCST). The LCST transition is mainly characterized by a drastic change in the interactions between water molecules and the hydrophilic region of the polymer due to hydrogen bonding and hydrophobic interactions between the polymer chains [132–134]. The phase transition of the polymer could lead to the controlled release of the loaded drug from the DDSs.

Karakasyan et al. condensed the polyetheramine group (PEA) into alginates that produced thermoresponsive alginate-block polyetheramine copolymer microgels (60-80 *μ*m) [134], corresponding to a propylene oxide/ethylene oxide ratio (PO/EO) of 29/6. They found a 10-20% reduction in the size of the microgels when the temperature was increased above the association temperature of the polymer, which not only demonstrated the thermosensitivity but also suggested it is caused by the expulsion of water from the microgels. Using ionic self-association between alginate and a monocationic copolymer (polyether amine, Jeffamine®-M2005) [135], several thermosensitive polyelectrolyte complexes were successfully prepared. It was suggested that electroassociation must be established below the LCST of the free Jeffamine®. The organization of the complexes is controlled by the thermoassociation of Jeffamine® previously electroassociated with alginate. Alginate-grafted poly(N-isopropylacrylamide) hydrogels (Alg-g-P(NIPAAm)) are used to locally deliver DNA nanoparticles for the treatment of castrate-resistant prostate cancer [136]. Six different Alg-g-P(NIPAAm) hydrogels were synthesized with 10% alginate and 90% NIPAAm; the result of which not only produced hydrogels with high molecular weight or low M/G ratio alginate backbone with greater stiffness. Furthermore, Alg-g-P(NIPAAm) hydrogels loaded with DNA nanoparticles also demonstrated suitable properties and were injectable at 20°C and solidified under physiological conditions.

#### 4.3.2. pH-Responsive Targeting

Numerous pH-sensitive delivery systems have been most widely used in cancer therapy. It is well known that pH values vary significantly in different organs or tissues, such as the stomach and colon, and disease states, such as inflammation, infection, and tumorigenesis [137]. Due to the high rate of glycolysis in cancer cells, the pH in tumors (5.7-7.0) is lower than that in healthy tissues (approximately 7.4) [137, 138]. At the subcellular level, even more significant pH differences were observed. Late endosomes and lysosomes have much lower pH (4.5-5.5), which is important for pH-sensitive DDSs design, since carriers and drugs are usually internalized through endocytosis and trapped within endosomal and lysosomal compartments [139].

Manatunga et al. produced curcumin and 6-gingerol-loaded pH-sensitive sodium alginate and hydroxyapatite bicoated iron oxide nanoparticles (IONP/HAp-NaAlg) (9.6 nm to 20 nm) for anticancer therapy [97]. pH-sensitive laponite/DOX/alginate (LP/Dox/AG) nanohybrids (142 ± 4 nm) were prepared with an encapsulation efficiency of 80.8 ± 10.6% to improve anticancer efficacy [103]. At physiological pH (pH 7.4), the cumulative release of DOX from the system was 6.2 ± 0.5% within 1 day, while under acidic pH conditions that resembled the extracellular environment of a solid tumor (pH 6.5) and the endolysosome internal milieu (pH 5.0), the DOX release rate was significantly faster, indicating the pH-sensitive drug release characteristics. *In vitro* studies showed that the LP/Dox/AG nanohybrids could be effectively internalized by CAL-72 osteosarcoma cells, and exhibit remarkable higher cytotoxicity in cancer cells compared with free DOX. Electrostatic interaction between the positively charged materials and the negatively charged alginate produces a pH-sensitive polyelectrolyte complex. pH-sensitive alginate/chitosan/kappa-carrageenan (Alg/Cs/kC) microcapsules were developed for the colon-targeted release of 5-fluorouracil, with a loading rate of 36.24% [140]. At gastric pH (1.2), the cumulative 5-fluorouracil release percentage of the dual-layered Alg/Cs/kC microbeads was 7%. The release profiles were greatly much improved under simulated intestinal and colon conditions to achieve the colon-specific anticancer effects. Furthermore, modification or coating of alginate particles with pH-sensitive bonds or layers of biomaterials to form a pH-sensitive carrier could be a potential strategy for better controlling the release rates of encapsulated drugs.

#### 4.3.3. Redox-Responsive Targeting

Since tumor tissues have high oxidative stress, they possess more glutathione (GSH) to tackle oxidative species especially in the intracellular environment [141, 142]. Redox-responsive DDSs are considered efficient for tumor targeting because of the significant difference in GSH concentrations between tumors and normal tissues. Moreover, redox-responsive DDSs are optimal for tumor intracellular delivery, because the intracellular concentration of GSH (approximately 2-10 mM) was significantly higher than that in the extracellular environment (2-20 *μ*M) [141, 143].

Sun et al. employed a simple crosslinking method to prepare DOX-loaded GSH/trypsin-responsive nanogels (DOX@KSA-NGs) (~100 nm) from human hair keratin and alginate [101]. The cysteine- and sulfhydryl-rich structure of human hair keratin makes it capable of responding to GSH. The DOX loading rate of the delivery system was 52.9 wt%. *In vitro* studies suggested that DOX@KSA-NGs were efficiently internalized in 4T1 and B16 cells, with a fast release of DOX into cells under intracellular GSH and trypsin levels. *In vivo* experiments showed that DOX@KSA-NGs had a better antitumor effect and lower side effects compared with free drugs. Yuan et al. also used DOX as a model drug to fabricate redox and pH dual-responsive nanocarriers based on sodium alginate end-capped mesoporous silica nanoparticles (MSN-SS-SA/DOX) (∼100 nm) [98]. The amount of sodium alginate grafted on the surface of the nanoparticles was approximately 46.12 mg/100 mg SiO_2_. Since sodium alginate shells coated the surface of nanoparticles by disulfide bonds, it could be shed and modulated the diffusion of loaded DOX in the presence of GSH. Moreover, the sodium alginate shell underwent a distinct transition from pH 7.4 to pH 5.0, which led to the release of DOX in the acidic tumor microenvironment. *In vitro* anticancer studies confirmed that MSN-SS-SA/DOX inhibited the growth of HeLa cells much more efficiently than free DOX. Herein, both nanogels and nanoparticles exhibited a very high loading efficiency for DOX, which could be a means for soluble anticancer drug delivery.

#### 4.3.4. External Physical Stimuli-Responsive Targeting

Cancer-targeted DDSs can be designed based on controlled release triggered by other parameters beyond the inner body, which are termed as “external stimuli,” including magnetic fields, ultrasound, and light. Compared with internal conditions in the cancer microenvironment, external triggers provide better controllable features for the release of the loaded drugs [144].

*(1) Ultrasound-Responsive Targeting*. Ultrasound, especially high-intensity focused ultrasound, is one of the largest application areas for exogenously triggered release owing to its noninvasiveness, ease of accessibility, controllable spatiotemporal effect, and high patient acceptability [129, 145, 146]. Baghbani and Moztarzadeh prepared DOX/curcumin-loaded alginate-shelled ultrasound-responsive phase-shift perfluorocarbon (PFC) nanodroplets (~55.1 nm) via a nanoemulsion process [104]. The entrapment efficiencies for DOX and curcumin were 92.3% and 40%, respectively. Alginate was coated on surfaces to cover and stabilize the PFC nanodroplets that could release their cargos locally in the target tissue under the action of ultrasound. It was found that the active drug release process was strongly correlated to the sonication frequency and low-frequency sonication resulted in enhanced acoustic cavitation and eventually higher ultrasound-induced drug release. *In vitro* studies indicated that sonication at a frequency of 28 kHz significantly enhanced the cytotoxicity of nanodroplets on A2780 human ovarian cancer cells. *In vivo* ovarian cancer treatment using nanodroplets combined with ultrasound irradiation resulted in efficient tumor regression. The authors further developed DOX-loaded ultrasound-responsive alginate/PFC nanodroplets (~51.7 nm) via the same nanoemulsion process [105]. The alginate shell could improve stealth properties from the reticuloendothelial system (RES) and result in higher accumulation of the drug at the tumor site through the EPR effect. The encapsulation efficiency of DOX in the nanodroplets was 93.8 ± 3.1%. *In vivo* therapy, using breast cancer models combined with sonication resulted in strong tumor regression efficiency. DOX concentration in the tumor area for the nanodroplet-treated group reached 10.9 *μ*g/g after sonication (28 kHz, 0.034 W/cm^2^), which was 5.2-fold higher compared with the nonsonicated nanodroplets group. In both studies, PFC nanodroplets can easily convert into microbubbles under the action of ultrasound, which results in the release of encapsulated drugs and enhanced intracellular uptake.

*(2) Magnetic Field-Responsive Targeting*. Magnetic field-responsive DDSs have also emerged as attractive therapeutics for cancer diagnosis and treatment. Generally, a magnetic field frequency below 400 Hz is hardly absorbed by the body and can be remotely directed to the desired tissue [147]. Magnetite (Fe_3_O_4_) or maghemite (Fe_2_O_3_) cores contained in DDSs can act as transducers to convert external electromagnetic energy into thermal energy, which could disrupt chemical bonding or change polymer characteristics (permeability, swelling, solubility, rigidity, and among others) in DDSs and control the release of the loaded drug [99]. Jardim et al. designed magneto-responsive MnFe_2_O_4_ nanoparticles functionalized with the layer-by-layer assembly of sodium alginate as a polyanion and chitosan as a polycation [99]. Curcumin-loaded platforms (~200 nm) with ~12 nm homogeneously embedded MnFe_2_O_4_ nanoparticles were obtained. *In vitro* cytotoxicity assays on human breast tumor cells (MCF-7) showed that entrapped curcumin could be remotely delivered and, upon application of an alternating magnetic field, its release could be controlled to specific targets. In this report, the layer-by-layer (LbL) deposition technique was also used, as it holds enormous potential for the development of stimuli-responsive alginate and chitosan due to its unique control of thickness and composition at the nanoscale. Moreover, drugs can be loaded between layers to produce a diversity of compositions and drug release profiles, and it may likewise increase control over when and where the drug is released through magnetic stimuli.

## 5. Conclusion

It is well known that the shortcomings of many anticancer agents, including off-target effects, undesirable biodistribution, and low therapeutic efficacy, have limited their clinical applications. To some extent, DDSs have been revolutionized by nanotechnology, microsphere techniques, and so on in the last decades, including cancer-targeted drug delivery with the primary purpose of maximizing the therapeutic efficiency and minimizing side effects. With a great deal of the free hydroxyl and carboxyl groups in the molecular chain, alginate as an ocean-sourced natural polymer could be easily modified with certain groups or ligands to obtain cancer targeting functionality. Advancements in technologies based on alginate have helped various medicines to obtain the properties mentioned above, such as decreased toxicity, increased bioavailability, and improved absorption. The joint use of alginate and its derivatives in various drug delivery technologies is promising for speeding up the process of cancer treatment, as well as protecting encapsulated drugs from degradation. Therefore, it is widely expected that the use of alginate in cancer-targeted DDSs will improve the prospects of pharmaceutical and biotechnology industries in the future. As reviewed above, alginate-based platforms are highly promising carriers for efficient drug delivery to cancer sites.

## Figures and Tables

**Table 1:** Nanosized alginate-based cancer-targeted drug delivery systems.

Nanocarriers (composition)	Particle diameters	Strategies of cancer targeting	Loaded drugs	Refs.
Nanoparticles (alginate/chitosan)	212-552 nm	Enhanced cellular uptake	Curcumin diglutaric acid	[91]
Nanoparticles (alginate/chitosan)	~80 nm	Enhanced cellular uptake	Doxorubicin	[92]
Nanoparticles (PLGA/alginate/chitosan)	Below 200 nm	EPR effect	Doxorubicin	[93]
Nanoparticles (PLGA/alginate/chitosan)	200 nm	Folic acid receptor-based endocytosis		[94]
Nanoparticles (alginate/chitosan)	115 nm	Folic acid receptor-based endocytosis	5-Aminolevulinic acid	[95]
Nanoparticles (alginate)	274.2 nm	Glycyrrhetinic acid-mediated endocytosis	Doxorubicin	[96]
Nanoparticles (iron oxide/alginate/hydroxyapatite)	9.6-20 nm	pH-responsive	Curcumin and 6-gingerol	[97]
Nanoparticles (mesoporous silica/alginate)	∼100 nm	Redox and pH dual-responsive	Doxorubicin	[98]
Nanoparticles (MnFe_2_O_4_/alginate/chitosan)	~200 nm	Magneto-responsive	Curcumin	[99]
Nanogels (alginate-cyclodextrin)	~55.1 nm	Pressure-sensitive	5-Fluorouracil	[31]
Nanogels (alginate)	~250 nm	Folic acid receptor-based endocytosis	Doxorubicin	[100]
Nanogels (alginate/keratin)	~100 nm	GSH/trypsin-responsive	Doxorubicin	[101]
Micelles (alginate-graft-poly(N-isopropylacrylamide))	30-300 nm	pH, ionic strength, or temperature-sensitive	5-Fluorouracil	[77]
Micelles (alginate-curcumin conjugate)	200 nm	Enhanced cellular uptake	Curcumin	[79]
Micelle (alginate-curcumin conjugate)	235 nm	ASGPR-mediated endocytosis	Curcumin	[78]
Liposomes (alginate-cisplatin conjugate)	110 nm	Epidermal growth factor receptor-mediated endocytosis	Cisplatin	[102]
Nanohybrids (alginate-doxorubicin conjugate)	~142 nm	pH-responsive	Doxorubicin	[103]
Nanodroplets (alginate)	~55.1 nm	Ultrasound-responsive	Doxorubicin/curcumin	[104]
Nanodroplets (alginate)	~51.7 nm	Ultrasound-responsive	Doxorubicin	[105]
Nanocomplexes (alginate/chitosan)	70-120 nm	Folic acid receptor-based endocytosis	Temozolomide and doxorubicin	[106]

## Data Availability

The data supporting this review are from previously reported studies and datasets, which have been cited.
